# Interpolation with Specified Error of a Point Series Belonging to a Monotone Curve

**DOI:** 10.3390/e23050493

**Published:** 2021-04-21

**Authors:** Yevhen Havrylenko, Yuliia Kholodniak, Serhii Halko, Oleksandr Vershkov, Larysa Bondarenko, Olena Suprun, Oleksandr Miroshnyk, Taras Shchur, Mścisław Śrutek, Marta Gackowska

**Affiliations:** 1 Department of Information Technologies of Design named after V. M. Naidysh, Dmytro Motornyi Tavria State Agrotechnological University, 18 B.Khmelnytsky Ave, 72312 Melitopol, Ukraine; yevhen.havrylenko@tsatu.edu.ua (Y.H.); yuliya.kholodnyak@tsatu.edu.ua (Y.K.); serhii.halko@tsatu.edu.ua (S.H.); oleksandr.vershkov@tsatu.edu.ua (O.V.); larysa.bondarenko@tsatu.edu.ua (L.B.); olena.suprun@tsatu.edu.ua (O.S.); 2 Department of Electricity and Energy Management, Kharkiv Petro Vasylenko National Technical University of Agriculture, 19 Rizdviana Str., 61052 Kharkiv, Ukraine; 3 Department of Cars and Tractors, Faculty of Mechanics and Energy, Lviv National Agrarian University, 1 Volodymyr Great Str., 80381 Dubliany, Ukraine; shchurtg@gmail.com; 4 Faculty of Telecommunications, Computer Science and Electrical Engineering, UTP University of Science and Technology in Bydgoszcz, 85-796 Bydgoszcz, Poland; mscislaw.srutek@utp.edu.pl (M.Ś.); marta.gackowska@utp.edu.pl (M.G.)

**Keywords:** monotone curve, tangent circle, adjacent circle, area of location of the curve, contour

## Abstract

The paper addresses the problem of modeling a smooth contour interpolating a point series belonging to a curve containing no special points, which represents the original curve with specified accuracy. The contour is formed within the area of possible location of the parts of the interpolated curve along which the curvature values are monotonously increased or decreased. The absolute interpolation error of the point series is estimated by the width of the area of possible location of the curve. As a result of assigning each intermediate point, the location of two new sections of the curve that lie within the area of the corresponding output section is obtained. When the interpolation error becomes less than the given value, the area of location of the curve is considered to be formed, and the resulting point series is interpolated by a contour that lies within the area. The possibility to shape the contours with arcs of circles specified by characteristics is investigated.

## 1. Introduction

Modeling is an effective tool for investigating objects, phenomena, and processes. Geometric modeling of an object often determines its functional properties. Such objects are, first of all, items bounded by functional surfaces which ensure the laminar nature of the item’s flow around by the environment–gas, liquid, or loose materials [1,2,3]. Examples of items limited by functional surfaces are automobile and aircraft hulls, working bodies of agricultural machinery, impeller blades of turbines, and compressors.

Complex surfaces are typically modeled based on linear frameworks whose elements are formed by interpolation of point series. The operating performance of the item is ensured by the geometric characteristics of the interpolated curves.

The improved aero- and hydrodynamic properties of the surface ensure the use of lines with regular variations in the values of characteristics and a minimum number of special points according to the statement of the problem as elements of the framework [4,5]. For a plane curve, these are the junction points of the convex and concave parts and the points where the curvature values are extreme. A smooth, plane curve that contains no special points will be referred to as a curve with a monotone curvature change or a monotone curve.

If a surface is modeled with the aim of creating a copy of an existing item (reverse engineering), the task of interpolating a point series becomes even more complex. In this case, the interpolation accuracy requirement is added to the requirement of ensuring the necessary characteristics of the interpolated curve [6].

In order to solve the stated problem, it is necessary to develop an interpolation method which ensures control of the change pattern of its characteristic values along the modeled curve, the possibility of local correction of the resulting solution, and prevention of uncontrolled emergence of special points.

The possibility of local correction of the shape of the interpolated curve while controlling its characteristics at output points is provided by methods of interpolation of the point series with a contour. The contour is formed by sections of analytically defined curves, which are connected at output points with a specified order of smoothness [7]. These are methods of interpolation by sections of second-order curves, Bézier curves, and B-splines [8,9,10,11,12].

Among the methods of interpolation of the point series with a contour, B-spline interpolation provides the greatest possibilities to adjust the sections of the contour.

A spline is defined by a set of control points, each of which has a transition function. It is a composite curve, each segment of which defines a separate equation. The curve approximates the broken line connecting the control points. The configuration of this broken line makes it possible to control the presence of inflection points in the B-spline.

The main disadvantage of B-spline interpolation as well as interpolation with other analytically defined curves is the lack of mechanisms to control the occurrence of points with extreme curvature. This disadvantage reduces the possibilities of using analytically defined curves while modeling shapes with the given characteristics and, above all, contours designed for modeling surfaces with specified functional properties.

Papers [7,9] solve the problem of interpolation of a point series by a contour consisting of smoothly joined arcs of circles. When each section is formed by an arc of one circle, local adjustment of the contour is not possible. The change of the radius of any of the arcs changes the configuration of all the sections of the contour. The problem is solved by forming sections with two or more arcs of circles at fixed positions of tangents to the contour at output points. In this case, adjustment of an individual section does not imply a reconfiguration of other sections of the contour.

The problem of forming a contour along which the radii of the arcs of circles change monotonically as well as the problem of ensuring the specified interpolation accuracy is not considered in [7,9].

The aim of the study is to develop a method for forming a smooth contour interpolating a point series that represents a monotone curve with specified accuracy.

In order to achieve this aim, the following objectives should be pursued:

- develop a method for forming the area of the monotone curve interpolating a given point series whose width does not exceed a specified value;
- to develop a method for forming a smooth contour consisting of arcs of circles, which interpolates the given point series and is located within the area of the monotone curve;
- to investigate the possibilities of the proposed method in solving the problem of interpolation of a given point series.

## 2. Materials and Methods

Formation of the area of location of the monotone curve is illustrated by the point series, which can be interpolated by a curve with a monotonous increase in the radii of curvature. Let us consider the following tasks:

- analyzing a output point series, which makes it possible to identify its parts that can be interpolated by a monotone curve;
- determining the absolute interpolation error of a point series by a monotone curve.

The analysis of the output point series is based on determination of the radii of adjacent circles (AC), each of which passes through three consecutive points in the series. The part of the point series along which the range of AC increases or decreases may be interpolated by a monotone curve along which the radii of curvature increase or decrease accordingly.

The absolute interpolation error of a section of a point series of a monotone curve is determined by the width of the area bounded by arcs of the corresponding AC ($\Delta_{i}^{AC}$).

**Figure 1:**
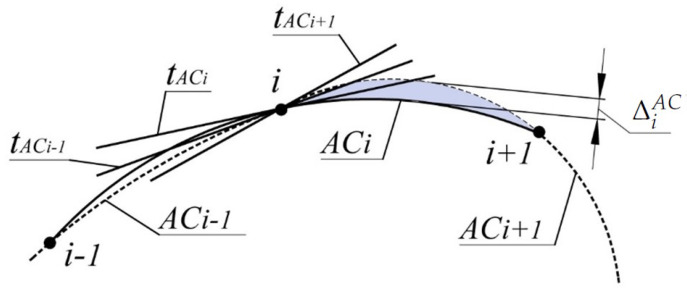
Area of location of the monotone curve.

For section (i,i+1), this area is bounded by the arc $AC_{i}$, which passes through the points i−1, *i*, i+1 and the arc $AC_{i+1}$ (Figure 1).

If the positions of the tangents $t_{i}$, $t_{i+1}$ and the radii of curvature $R_{i}$, $R_{i+1}$ are known at the points of the monotone curve *i* and i+1, then the absolute error of interpolation of its sections can be determined more precisely.

**Figure 2:**
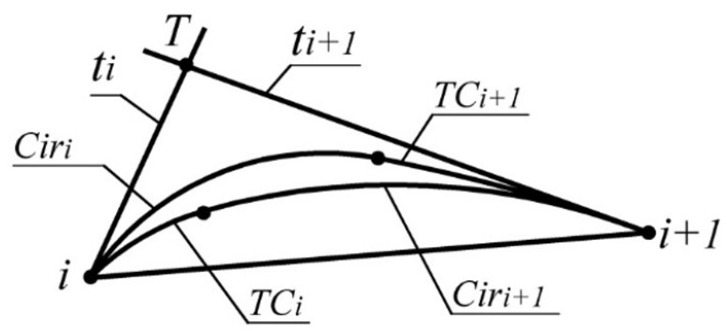
Refinement of the area of location of the monotone curve.

It is established that, for lines $t_{i}$ and $t_{i+1}$ tangent to the monotone curve along which the radii of curvature increase monotonously, the distances from the intersection point of the lines (pointT) to the points of tangency with the curve (Figure 2) correlate as

(1) |i,T|<|T,i+1|

Correlation (1) can be used as a criterion for the correct assignment of tangent lines in the formation of a contour with specified accuracy representing the monotone curve. For section of the curve (i,i+1), the error is estimated by the maximum distance between the two boundaries (Figure 2):

- a boundary consisting of the arc of the tangent circle at point *i* $\left(TC_{i}\right)$ and the arc of the circle tangent to $TC_{i}$ and line $t_{i+1}$ at point $i+1\;(Cir_{i+1})$. For the curve along which the radii of curvature increase, this limit of the area is lower (closest to the chord [i,i+1]);
- a boundary consisting of the arc of the tangent circle at point i+1 $\left(TC_{i+1}\right)$ and the arc of the circle tangent to $TC_{i+1}$ and line $t_{i}$ at point *i* $\left(Cir_{i}\right)$.

If the values of absolute interpolation error are greater than the given value, more interpolated curve points specifying the interpolated curve must be assigned to the corresponding sections of the original curve.

If the values of absolute interpolation error $\Delta_{i}^{AC}$ are greater than the given value, more interpolated curve points specifying the interpolated curve must be assigned to the corresponding sections of the original curve.

The final solution can be represented in the form of an accompanying broken line, the distance from which to the curve with the specified geometric characteristics does not exceed a predetermined value. The use of contours consisting of straight line sections in the modeling of functional surfaces reduces their aero- and hydrodynamic properties [7]

Let us define the constraints on the location of the section of the curve located between two consecutive output points.

The most accurate estimate of the area of the monotone curve is possible when the position of the centers of curvature $C_{i}\;and\;C_{i+1}$ for points iandi+1 is known. To define the boundaries of the area, the positions of tangent lines to the monotone curve at output points are predefined. Positions of the tangents must meet condition (1). One option for determining the necessary location of the tangents is to assign them within the ranges limited by AC at output points. For *i* point of the series, the range is bounded by the tangent to ${AC}_{i}$$\left(t_{ACi}\right)$ and the tangent nearest to it ${AC}_{i-1}\;and\;{AC}_{i+1}$ (Figure 1).

Once the tangent to the monotone curve has been assigned at each of the output points, the upper and lower boundaries of the area of possible location of the monotone curve interpolating the point series are determined.

**Figure 3:**
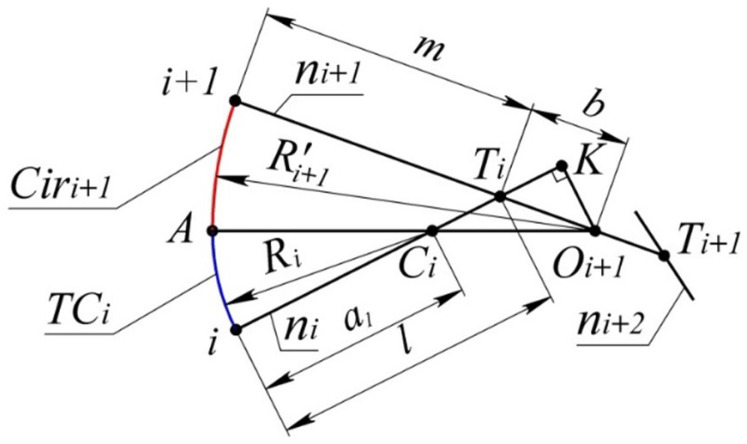
Lower boundary of the area of location of the section of the monotone curve.

The lower boundary of the area is represented by the arc $TC_{i}$ and the arc of the circle $Cir_{i+1}$ tangent to the curve at point i+1, and with $TC_{i}$ at some point *A* (Figure 3).

The given data for determining the lower boundary of the area are the position of the normal of the curve $n_{i}$ and $n_{i+1}$ at points *i* and i+1, and the position of the center of curvature $C_{i}$.

The problem reduces to determining the position of the center of $Cir_{i+1}$ (point $O_{i+1}$). Let us introduce the following notations to be used for the problem: $T_{i}$—the point where normals $n_{i}$ and $n_{i+1}$ intersect (Figure 3); $T_{i+1}$—the point where normals $n_{i+1}$ and $n_{i+2}$ intersect; $b=|T_{i},T_{i+1}|;\;a_{1}=|C_{i},T_{i}|;\;m=|i+1,T_{i}|,$ $S_{i}$ - the area of the triangle $C_{i}$, $T_{i}$, $T_{i+1}$.

The position of the center of $Cir_{i}$ within the segment $\left[T_{i},T_{i+1}\right]$ is unambiguously determined by the correlation:

(2) $$M_{i+1}=\frac{|T_{i},O_{i+1}|}{b}\;$$

Based on the tangency of the arcs i−A and A−i+1:

(3) $$|C_{i},O_{i+1}|=m-R_{i}+M_{i+1}\cdot b$$

Based on the triangle $C_{i}$, $O_{i+1}$*K*:

(4) $$|C_{i},O_{i+1}{|}^{2}=|K,C_{i}{|}^{2}+|K,O_{i+1}{|}^{2}=a_{1}^{2}+2a_{1}\left|T_{i},K\right|+M_{i+1}^{2}\cdot b^{2}$$

The length of the segment $\left[T_{i},K\right]$ shall be determined as a leg of the triangle $T_{i},K,O_{i+1}:|T_{i}{,K|}^{2}=M_{i+1}^{2}\cdot b^{2}-{\left|K,O_{i+1}\right|}^{2}$.

The length of the segment $\left[K,O_{i+1}\right]$ shall be expressed through the area $\left(S_{1}\right)$ of the triangle $C_{1},O_{i+1},T_{i}$ as $|K,O_{i+1}|=\frac{2S_{1}}{a_{1}}$.

By expressing $S_{1}=MS_{i}$, let us bring expression (4) to:

(5) $$|C_{i},O_{i+1}{|}^{2}=a_{1}^{2}+2M_{i+1}\sqrt{a_{1}^{2}b^{2}-4S_{i}^{2}}+M_{i+1}^{2}\cdot b^{2}$$

Having plugged (3) into (5) after transformations, we obtain:

(6) $$M_{i+1}=\frac{a_{1}^{2}-{\left(m-R_{i}\right)}^{2}}{2(b\left(m-R_{i}\right)-\sqrt{a_{1}^{2}b^{2}-4S_{i}^{2}})}\;$$

The upper boundary is formed by the arc $TC_{i+1}$ and the arc of the circle $Cir_{i}$, which is tangent to the curve at point *i*, and with $TC_{i}$ at some point B.

The problem of determining the upper boundary of the area of the monotone curve is reduced to calculating the coordinates of the center of $Cir_{i}$ (point $O_{i}$). The given data for the problem are the position of the normals $n_{i},n_{i+1},n_{i+2}$ and the center of curvature $C_{i+1}$.

**Figure 4:**
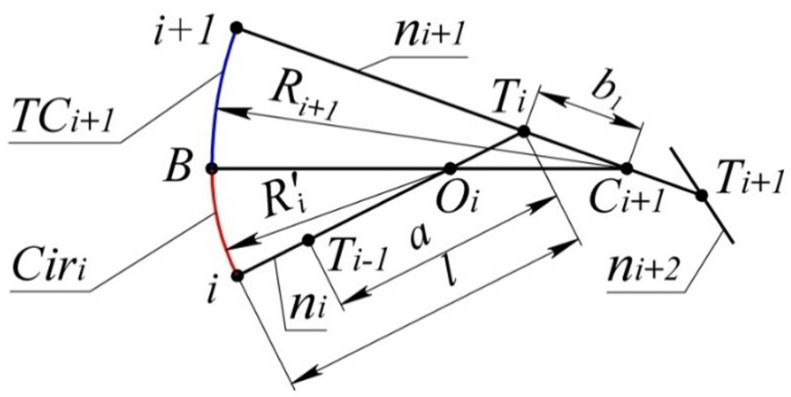
Upper boundary of the area of location of the section of the monotone curve.

The position of $O_{i}$ within the segment $\left[T_{i+1},T_{i}\right]$ (Figure 4) is determined by the correlation:

(7) $$M_{i}=\frac{|O_{i},T_{i}|}{a}\;$$

where a = $|T_{i+1},T_{i}|$.

As a result of manipulations similar to those done in the derivation of expression (6), we obtain:

(8) $$M_{i}=\frac{b_{1}^{2}-{\left(R_{i+1}^{2}-l\right)}^{2}}{2(a\left(R_{i+1}-l\right)-\sqrt{a^{2}b_{1}^{2}-4S_{i+1}^{2}})}\;$$

where $l=|i,T_{i}\left|,\;a=\right|T_{i+1},T_{i}|,\;b_{1}=\left|T_{i},C_{i+1}\right|$, and $S_{i+1}$ is the area of the triangle $T_{i+1},T_{i},C_{i+1}$.

At the specified position of normals $n_{i},n_{i+1}$ and the center of curvature $C_{i+1}$, the radius of $Cir_{i}$ is the maximum possible radius of curvature of the monotone curve at point *i*. Similarly, the position of $C_{i}$ determines the minimum possible radius of curvature at point $i+1-R_{i+1}^{\prime }=\left|O_{i+1},i+1\right|$.

Based on this criterion, let us determine the area of the monotone curve, driven by a random point series.

**Figure 5:**
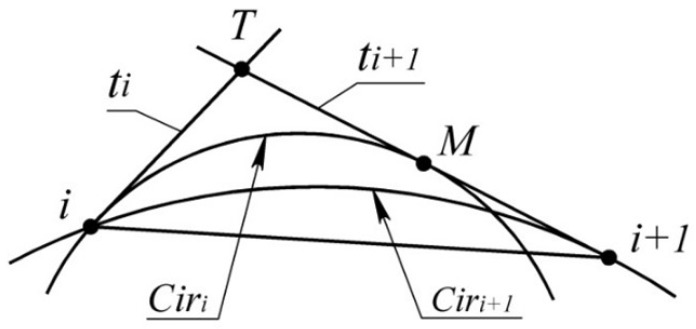
Finding possible values of the radii of curvature.

Provided that the radii of curvature along the curve increase monotonously, the minimum radius of curvature at point *i*$R_{i}^{min}$ equals zero. Here, point i is considered to be the tangent circle of zero radius. In this case, the lower boundary of the interpolated curve is the arc of circle $Cir_{i+1}$, which is tangent to line $t_{i+1}$ at point i+1 and passes through point i (Figure 5). The minimum possible radius of curvature that can be assigned at point i+1, $\left(R_{i+1}^{min}\right)$ is equal to the radius of $Cir_{i+1}$.

The maximum radius of curvature that can be assigned at point i+1$\left(R_{i+1}^{max}\right)$ is equal to infinity. In this case, the upper boundary of the area of location of the formed curve is the curve consisting of the arc of the circle tangent to $T_{i}$ at point *i* and to $t_{i+1}$ at point *M* $Cir_{i}$ and the segment [M;i+1] (the tangent circle of infinite radius). At point *i*, the maximum possible radius of curvature $\left(R_{i}^{max}\right)$ equals the radius of $Cir_{i}$.

All curves with monotonous increase of the radii of curvature, having at points *i* and i+1 tangents $t_{i}$ and $t_{i+1}$ respectively, pass within the area bounded by the resulting composite curves. The radii of curvature of the curve satisfying the conditions of the problem at points *i* and i+1 must belong to the following ranges:

(9) $$0\leq R_{i}\leq R_{i}^{max}\;and\;R_{i+1}^{max}\leq R_{i+1}\leq \infty$$

If a monotone curve interpolates a sequence of points 1…n, then the minimum radius of curvature can be equal to zero only at the first point, and the maximum radius can be equal to infinity only at the last point.

In this case, the lower boundary of the area in which the curve is located shall be determined in the following way.

1. In sections 1 and 2, the area is bounded by the arc of the circle passing through point 1 and having a common tangent to the curve at point 2. The specified circle is taken as the tangent circle at point 2, whose radius is the minimum possible. Let us mark this circle as $TC_{2}^{min}$
2. Taking $TC_{2}^{min}$ as the tangent circle at point 2, we determine the circle tangent to it and to the monotone curve at point 3. Let us denote this circle $TC_{3}^{min}$. The position of the center shall be determined by the procedure presented in Figure 3 for point $O_{i+1}$. The lower boundary of the area of location of the curve in sections 2 and 3 is formed by smoothly conjoined arcs of circles $TC_{2}^{min}$ ∦ $TC_{3}^{min}$
3. Based on the location and the size of $TC_{3}^{min}$, the lower boundary of the area in sections 3 and 4 is similarly determined, as well as the boundaries of the remaining sections.

The upper boundary of the area of location of the monotone curve is determined starting with the last section by the following scheme.

1. In section (n−1,n), the boundary of the area consists of the tangent line segment at point *n*$\left(t_{n}\right)$ and the arc of circle $TC_{n-1}^{max}$, which is tangent to $t_{n}$ and has a common tangent to the curve at point n−1. The specified circle is taken as the tangent circle at n−1, whose radius is the maximum possible.
2. In section (n−2,n−1), the upper boundary of the area consists of the arcs of circles $TC_{n-1}^{max}$ and $TC_{n-2}^{max}$ tangent to it which has a common tangent to the monotone curve at point n−2. The center of $TC_{n-2}^{max}$ shall be defined by the procedure presented in Figure 4 for point $O_{i}$.
3. The upper boundary of the area of location of the curve is determined in sequence from section to section, similarly to the boundary of section (n−2,n−1).

**Figure 6:**
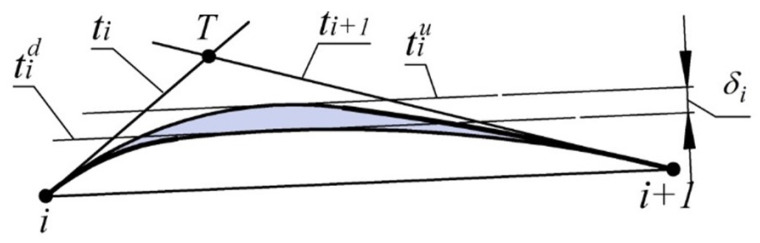
Finding the absolute interpolation error.

The absolute error of interpolation $\left(\delta_{i}\right)$ of a point series by a monotone curve is estimated by the width of its possible location. In section i…i+1, the width of the area shall be defined as the distance between lines $t_{i}^{u}$ and $t_{i}^{d}$, which are parallel to segment [i,i+1] and are tangent to the lower and upper boundaries of the area respectively (Figure 6).

If the interpolation error of the output point series is greater than the assigned value, the width of the area of location of the curve is reduced by assigning intermediate points belonging to it. The intermediate point shall be assigned to the line passing through the middle of segment [i,i+1], at right angles to this segment within the area of location of the curve. By assigning each intermediate point, we obtain the location of two new sections of the curve which lie within the area of the corresponding output section.

After the interpolation error becomes less than the specified value, the area of the monotone curve is considered to be formed, and the resulting point series is interpolated by a contour which lies within the area of location of the curve. It is appropriate to take the minimum possible processing error on a numerical control machine, which is $10^{-3}$ mm, as the value that cannot exceed the specified interpolation error [6].

To solve the posed problem, it is necessary to ensure the following:

- the presence of a common tangent to the contour and the monotone line curve at output points;
- the increase of the radii of curvature along the contour in the same direction as the monotone curve.

Let us consider the possibility of forming contours with specified characteristics of the arcs of circles.

**Figure 7:**
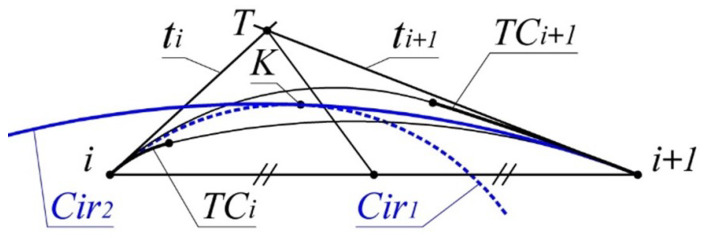
Replacement of the monotone curve with arcs of circles.

The formation of a contour consisting of two arcs of circles which replace section [i,i+1] with a monotone curve along which the radii of curvature monotonously in-crease is represented in Figure 7.

The circle defining the first arc $\left(Cri_{1}\right)$ is driven by a tangent with a monotone curve at point *i* and passing through some point assigned within the area of location of the monotone curve. For instance, this point may be the middle of a segment that is bounded by the intersection points of the median of triangle *i*, *T*, i+1 with the boundaries of the area of location of the monotone curve.

The circle to which the second arc $\left(Cri_{2}\right)$ belongs is defined by touching the monotone curve at point i+1 and by touching $\left(Cri_{1}\right)$ at some point *K*. As a result, we obtain a circle with the correlation of the radii:

(10) $$R_{i}^{min}<R_{1}<R_{i}^{max}\;and\;R_{i+1}^{min}<R_{2}<R_{i+1}^{max}\;and\;R_{1}<R_{2}$$

where $R_{1}$ and $R_{2}$-radii of $\left(Cri_{1}\right)$ and $\left(Cri_{2}\right)$, respectively; $R_{i}^{min},\;R_{i}^{max},\;R_{i+1}^{min},\;R_{i+1}^{max}$-radii of $TC_{i}^{min},\;TC_{i}^{max},\;TC_{i+1}^{min},\;TC_{i+1}^{max}$.

By similarly forming the arcs replacing the remaining sections of the monotone curve, we get a contour interpolating the whole point series along which the radii of the arcs of circles increase monotonously.

The monotonic increase of the radii of the circles along the contour in the same direction as the increase of the radii of curvature along the monotone curve, as well as the common tangents of the contour and the curve at output points, ensure the position of the contour within the area of location of the monotone curve.

The main drawback of forming the contour by arcs of circles is the irregular variation of the values of curvature at the points at which they are conjoined. Reducing the effect of the mentioned drawback on the functional characteristics of the surface while using the contour as an element of the framework is possible by increasing the number of arcs constituting the contour while reducing the difference between the values of the radii of the circles that determine them.

## 3. Results and Discussion

The possibilities of the method for shaping contours proposed in the paper are investigated by the example of interpolation of a sequence of ten points. The position of the specified points was determined based on the condition of a monotonous increase in the AC radii along the point series. The characteristics of the point series: coordinates of output points—$i(x_{i},y_{i})$; distance between adjacent output points—|i,i+1|; radii of AC defined by the point series—$R_{Aci}$; absolute error of interpolation of a point series by a monotone curve, specified by the coordinates of output points—$\Delta_{i}^{AC}$ are given in Table 1.

The monotonous increase of $RAC_{i}$ along the point series allows for interpolating it by the monotone curve along which the values of the radii of curvature increase. The absolute interpolation error is estimated as the maximum distance between the arcs $AC_{i}$ and $AC_{i+1}$, bounded by points *i* and i+1 (Figure 1). In the first and the last sections, the value $\Delta_{i}^{AC}$ is not defined as $AC_{1}$ and $AC_{10}$ do not exist.

The next step in formation of the contour at output points is denoting the position of the tangents to the monotone curve interpolating the point series and the refined area of its possible location at each of the sections. The positions of the tangents are defined within the ranges bounded by the corresponding AC (Figure 2). The length of the sides of triangles obtained from assigning the tangent lines–|i,i+1|, |i,T|, |i+1,T|; the absolute error of interpolation of a point series by a monotone curve driven by the coordinates of output points and the position of tangents at these points at each of the sections–$\Delta_{i}$; the exceedance of the lower boundary of the area of location of the interpolated curve over the segments, connecting the corresponding output points $h_{i}$, is given in Table 2.

The boundaries of the area of location of the interpolated curve are determined by the procedure presented in Figure 6. The absolute interpolation error in section i…i+1 is defined as the distance between the lines tangent to the upper and lower boundaries of the area of location of the curve and parallel to section [i,i+1].

The maximum absolute interpolation error was 1.1281 mm in section 9...10.

In order to reduce the maximum absolute interpolation error in reference section 9...10, an intermediate point and a tangent line to the monotone curve are assigned. The intermediate point is assigned within the area of location of the interpolated curve in section 9...10 on a line which passes through the middle of section [9,10] at right angles to segment B. As a result, we obtain a point series consisting of 11 points where the intermediate point is assigned No. 10. The position of the point is determined by the coordinates $x_{i}$= 168.62, $y_{i}$ = −2.92.

The characteristics of the area of possible location of the monotone curve interpolating the point series are given in Table 3.

For the obtained point series, the maximum absolute interpolation error was 0.2257 mm in section 8...9. By assigning intermediate points, it is possible to form a point series consisting of any number of points, which determines the arbitrarily small interpolation error of the monotone curve.

The characteristics of the contour consisting of smoothly joined arcs of circles interpolating a sequence of eleven points are given in Table 4.

Each of the sections of the contour consists of two arcs of circles of radius $R_{1}^{i}$ and $R_{2}^{i}$, accordingly, which possess a common tangent to the monotone curve at output points. The maximum exceedance of the section of the contour over the segment connecting the respective output points is indicated in the table as $H_{i}$. The absolute error with which the contour replaces the monotone curve $\delta_{i}$ is estimated by the maximum distance from the arcs of circles comprising the contour to the boundaries of the area of location of the corresponding sections of the monotone curve.

Formation of the sections of the contour by the procedure presented in Figure 7 ensured its location within the area of possible location of the monotone curve. The maximum absolute error with which the contour replaces the monotone curve was 0.2620 mm in section 8...9.

**Figure 8:**
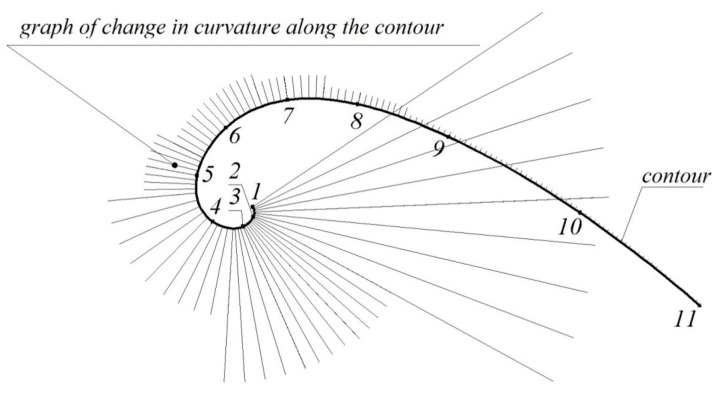
Graph of change in curvature along the contour.

Figure 8 represents the contour formed in the CAD system Solidworks, the characteristics of which are presented in Table 4. With the aid of the CAD system, the graph of change in curvature along the contour is formed. The resulting graph shows its monotonous change and irregular variation at joining points of the arcs of circles. The possibility of increasing the number of arcs forming the contour makes it possible to reduce arbitrarily the difference of the curvature values at their joining points.

The method proposed in the article was tested when creating a model of an impeller of an axial and radial flow turbocharger. The initial data for the design of the geometric model of the impeller were taken from a drawing designed for quality assurance of the finished product. The drawing contains tabulated data specifying an ordered array of 77 points belonging to the blade surface. The hub is driven by the axis of rotation and the generating line.

The model of the blade surface was designed on the basis of a framework, consisting of eleven plane sections. Each plane section was initially represented by seven points. Analysis of the original point series showed a monotone dynamics of changes in the values of the radii of the AC along each of them.

For example, the original point series representing the fourth section of the blade surface specifies the following radii of successive AC—23.65, 29.18, 40.37, 65.74 and 160.08 mm. The maximum error with which the original point series represents a monotone curve is $3.017\times 10^{-2}$ mm. The specified error is determined by the width of the band limited by a sequence of adjacent circles specified by a point series. As a result of assigning the positions of the intermediate points, a point series is obtained consisting of twenty-five points representing the horizontal section of the blade surface. At the points obtained, the positions of the adjacent circles of the monotone curve are assigned and the area of its possible location is determined. The error values with which the point series represents the sections of the monotone curve are within the range from $2\times 10^{-4}$ mm to $5\times 10^{-4}$ mm. The points are interpolated by the obtained compound curve of the circles.

**Figure 9:**
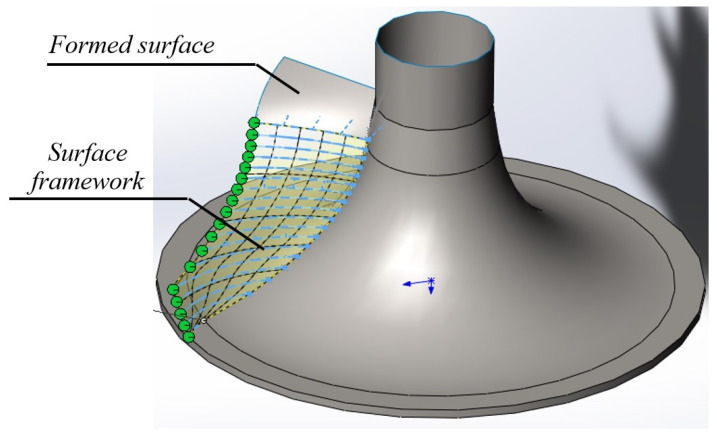
Model of the blade surface.

The eleven formed contours, each of which interpolates a point series consisting of twenty-five points, make up a family of generating lines of the framework of the blade surface model. The contours were imported into the CAD system SolidWorks and a model of the working surface of the blade was obtained using the “Loft Surface” function of the CAD system (Figure 9).

Based on the obtained frameworks of the hub and the blades, a CAD model of the compressor impeller was created. The resulting model was used as the given data for creating a program for processing the impeller surfaces on a CNC machine in the CAM system PowerMill. The model of the product was imported from the CAD system SolidWorks to the CAM system PowerMill is using direct data translators. The program control for NC manufacturing was created using the standard functions of the CAM system.

## 4. Conclusions

The problem of forming a smooth contour which interpolates a point series and represents a monotone curve with specified accuracy is solved in the paper. The following methods have been developed in order to achieve the objective:

- forming the area of possible location of the monotone curve interpolating a given point series the width of which does not exceed the given value;
- forming a smooth contour consisting of the arcs of circles which is located within the area of location of the monotone curve.

The area of possible location of the monotone curve is formed as closed contours which are joined at output points. The width of the area is determined by the maximum possible distance between the lines with the specified characteristics. All the monotone lines interpolating the point series are located within the area. The developed method provides an arbitrarily wide area by assigning intermediate points for the output point series.

The developed method for forming a contour within the area of the monotone curve ensures its replacement with a contour with an error which does not exceed the width of the area.

The possibilities of the proposed method have been investigated in solving the problem of interpolation of a point series consisting of 10 points using the CAD system SolidWorks. By solving the test case, it has been established that the minimum possible interpolation error is provided by a contour along which curvature values change monotonously and which has common tangents with the monotone curve at output points. The developed method for forming a contour by the arcs of circles meets the stated requirements.

The main field of use of the developed method is modeling of linear elements of frameworks of surfaces with improved aero- and hydrodynamic properties, including the use of reverse engineering.

The main disadvantage of the proposed method is that the curvature values at junction points of the sections of the contour are irregular. While solving applied problems, the effect of this disadvantage can be eliminated by increasing the number of sections. 

## Figures and Tables

**Table 1:** The characteristics of the point series.

*i*	$x_{1}$	$y_{1}$	|i,i+1|	$R_{\mathrm{Aci}}$	$\Delta_{i}^{\mathrm{AC}}$
1	0	0	5.28	-	-
2	0.21	−5.28	7.20	7.41	0.4966
3	−5.11	−10.13	15.57	13.30	0.9431
4	−20.48	−7.65	25.24	20.04	1.8869
5	−28.68	16.23	28.82	32.08	1.4296
6	−13.87	40.95	34.90	53.22	0.8614
7	17.95	55.28	36.29	74.21	1.1654
8	54.24	53.04	49.65	151.68	1.3118
9	100.94	36.19	155.96	420.54	–
10	230.38	−50.82	–	–	–

**Table 2:** The result in formation of the contour.

	BT				
i	|i,i+1|	|i,T|	|i+1,T|	$h_{i}$	$\Delta_{i}$
1	5.28	3.07	3.12	0.7327	0.0140
2	7.20	3.78	3.83	0.5906	0.0078
3	15.57	7.80	9.97	1.8639	0.0218
4	25.24	13.53	16.52	3.9238	0.0595
5	28.82	13.67	16.71	2.2022	0.0629
6	34.90	16.95	19.53	2.6298	0.0922
7	36.29	15.63	21.32	1.5500	0.2184
8	49.65	20.41	29.53	1.2163	0.2257
9	155.96	69.13	88.31	4.8396	1.1281

**Table 3:** The characteristics of the area of possible location.

	BT				
i	|i,i+1|	|i,T|	|i+1,T|	$h_{i}$	$\Delta_{i}$
1	5.28	3.07	3.12	0.7327	0.0140
2	7.20	3.78	3.83	0.5906	0.0078
3	15.57	7.80	9.97	1.8639	0.0218
4	25.24	13.53	16.52	3.9238	0.0595
5	28.82	13.67	16.71	2.2022	0.0629
6	34.90	16.95	19.53	2.6298	0.0922
7	36.29	15.63	21.32	1.5500	0.2184
8	49.65	20.41	29.53	1.2163	0.2857
9	78.16	37.03	41.39	1.5356	0.1753
10	78.16	37.63	40.65	1.0480	0.0975

**Table 4:** The characteristics of the contour.

*i*	$R_{1}^{i}$	$R_{2}^{i}$	$H_{i}$	$\delta_{i}$	$R_{1}^{i}$
1	4.9902	5.1701	0.7461	0.0134	4.9902
2	11.0191	11.3487	0.5962	0.0056	11.0191
3	11.4630	19.9296	1.8851	0.0212	11.4630
4	19.9359	34.1886	3.9828	0.059	19.9359
5	34.5156	53.4423	2.2646	0.0624	34.5156
6	53.5381	73.6735	2.7214	0.0916	53.5381
7	76.7344	145.9776	1.7641	0.2141	76.7344
8	163.8066	352.0208	1.4783	0.2620	163.8066
9	428.4307	537.2404	1.6269	0.0913	428.4307
10	660.3963	775.5817	1.0971	0.0483	660.3963

## Data Availability

The data presented in this study are available on request from the corresponding author.
